# Sub-GOFA: A tool for Sub-Gene Ontology function analysis in clonal mosaicism using semantic (logical) similarity

**DOI:** 10.6026/97320630018053

**Published:** 2022-01-31

**Authors:** Tadaaki Katsuda, Noriko Sato, Kaoru Mogushi, Takeshi Hase, Masaaki Muramatsu

**Affiliations:** 1 Department of Molecular Epidemiology, Medical Research Institute, Tokyo Medical and Dental University, 24F, M&D Tower, 1-5-45 Yushima, Bunkyo-ku, Tokyo, 113-8510, Japan; 2 Institute of Education, Tokyo Medical and Dental University, 20F, M&D Tower, 1-5-45 Yushima, Bunkyo-ku, Tokyo, 113-8510, Japan; 3 Faculty of Pharmacy, Keio University, 1-5-30 Shibakoen, Minato-ku, Tokyo, 105-8512, Japan; 4 The Systems Biology Institute, SaiseiIkedayama Bldg. 5-10-25 Higashi Gotanda Shinagawa, Tokyo, 141-0022, Japan; 5 SBX BioSciences, Inc, 1600 - 925 West Georgia Street, Vancouver, BC V6C 3L2, Canada

**Keywords:** Sub-GOFA, tool, sub-gene ontology, function, clonal mosaicism, semantic, logical, similarity

## Abstract

Clonal mosaicism (a detectable post-zygotic mutational event in cellular subpopulations) is common in cancer patients. Detected segments of clonal mosaicism are usually bundled into large-locus regions for statistical analysis. However, low-frequency genes are overlooked and are not sufficient to elucidate qualitative differences between cancer patients and non-patients. Therefore, it is of interest to develop and describe a tool named Sub-GOFA for Sub-Gene Ontology function analysis in clonal mosaicism using semantic similarity. Sub-GOFA measures the semantic (logical) similarity among patients using the sub-GO network structures of various sizes segmented from the gene ontology (GO) for clustering analysis. The sub-GO's root-terms with significant differences are extracted as disease-associated genetic functions. Sub-GOFA selected a high ratio of cancer-associated genes under validation with acceptable threshold.

## Background:

Clonal mosaicism is a post-zygotic large-scale mutational event in chromosomes and mitochondria in cellular subpopulation. It occurs in the peripheral blood with aging and in tissue, which are known to be associated with cancer [1- 9] and diabetes [10,11]. Genetic function analysis of rare clonal mosaics is generally performed by bundling them into large-locus regions for statistical analysis. Thousands of genes are affected under abnormal regions in clonal mosaicism, but a large number of genes in the regions are low-frequency genes that are uncommon among patients. Conventional statistical approaches overlook the effects of low-frequency genes and their genetic functions and are not sufficient to elucidate qualitative differences between cancer patients and non-patients. Recent advancements in high-throughput biological technologies have led to a significant accumulation of structured biological knowledge, and new approaches based on semantic technology are being attempted to exploit this information. The gene ontology (GO)[12] is one of the international projects that aims to create a common vocabulary in the field of life science research regarding the description of genetic functions. Each GO term is associated with the form of a hierarchical structure, which represents semantic relationships of inclusion. Using biological knowledge embedded in the GO structure has enabled further comparison or classification of given set of genes obtained by various omics analysis techniques (e.g., genomics, transcriptomics, and proteomics) to understand the biological phenomena? Currently, number of semantic-based tools has played an important role in improving analysis of proteomics and transcriptomics at the level of functional genomics using different semantic similarity measures among GO terms [13]. This approach has not yet been attempted for large-scale genomic regional dataset that consists of gene list in specific genomic region. The pair wise approach measures the individual semantic similarity for every pair of terms and integrates these into a global similarity measure. This global similarity measure is dependent on the size and structure of the network. Another important property of GO is a huge network structure of hierarchical directed acyclic graphs (DAGs). In clonal mosaicism, pair-wise semantic similarity measure between large-scale genomic regional datasets handles thousands of genes as variables. Even if the similarity of a characteristic genetic functions is found in a segmented specific GO network region, that similarity is homogenized within a global similarity measure in the entire GO network, and those genetic functions are overlooked.

We attempt to obtain pairwise similarity between large-scale genomic regional datasets on patients using sub-graph structures of GO networks in various sizes to implement a novel method for genetic functional analysis considering low-frequency genes and GO terms. Since GO is a hierarchical network of DAG structures, it can be segmented from higher GO terms to lower GO terms. Sub-GO is a hierarchical network of partial genetic functions segmented from GO. By applying GO terms under each sub-GO network, we prevent homogenization of characteristic pairwise similarities between large-scale genomic regional datasets on patients in a segmented specific GO network region. By statistically evaluating the pairwise similarity between those patients, we can measure the influence of the genetic function of the sub-GO on the subject disease. In addition, sub-GO networks include all the genes, especially low-frequency genes, in GO annotations associated with patients and thus may be useful for genetic functional analysis for low-frequency genes. Therefore, it is of interest to develop and describe a tool named Sub-GOFA for Sub-Gene Ontology function analysis in clonal mosaicism using semantic similarity.

## Materials and methods:

### Sub-GOFA algorithm:

**Figure 1:**
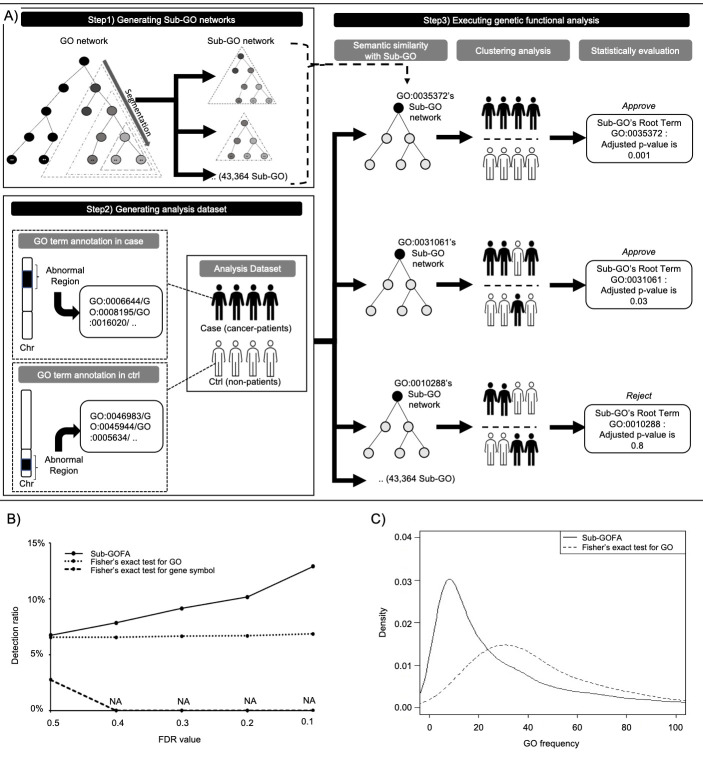
Sub-GOFA’s genetic functional analysis overview and performance for clonal mosaicism. A: The analysis flow of Sub-GOFA. Step 1 - Generating Sub-GO networks (43,364) by segmenting the huge GO network. Step 2 - Generating analysis dataset annotating multivariate GO terms in abnormal regions for case (cancer-patients) and ctrl (non-patients). Step 3 - Executing genetic functional analysis consists of semantic similarity analysis with Sub-GO (43,364), clustering analysis from the similarity results and statistical evaluation with adjusted p-values. B: Comparison of lung cancer associated gene contents ratio of Sub-GOFA and Fisher's exact test for gene symbols and for GO terms with varying FDR values. C: Plot of the frequency and density of GO terms calculated by Sub-GOFA and Fisher's exact test for GO in lung cancer analysis dataset at FDR value of 0.3.

Figure 1A shows an overview of the Sub-GOFA algorithm. First, Sub-GOFA segments the overall GO (version 1.2) network into 43,364 Sub-GO networks. Next, Sub-GOFA measures the information criterion (IC)-based semantic similarity between large-scale genomic regional datasets annotated with multiple GO terms for all patient pairs, including cancer patients and non-patients. Sub-GOFA adopted Lin's method [14] in ontology Similarity (version 2.5) [15], which were implemented in R statistical language version 3.4.3 (http://www.r-project.org/), for IC-based semantic similarity. Then, cluster analysis by the Ward's method is performed to classify all samples in a dataset into two groups based on the similarity results of all pairs of samples. Between those two groups, there may be a significant difference in the proportion of cancer patients and non-patients. In the case of those with significant differences, the Sub-GO networks could be regarded as genetic functions associated with the differences between cancer patients and non-patients. Lastly, the proportions of cancer-patients and non-patients in each cluster are statistically evaluated using the Fisher's exact test adjusted by the false discovery rate (FDR), and root terms of sub-GO with significant differences are extracted as disease-associated genetic functions. Sub-GOFA is not published, and thesource codes are available from the authors upon request.

### Clonal mosaicism dataset:

The clonal mosaicism dataset for Sub-GOFA was created using public domain datasets [1-6]. Each sample in this dataset describes the clinical phenotype information of the specific cancer name, non-disease, or other-disease name and the position information of the abnormal region of the clonal mosaicism, such as chromosome number, start position, and end position. The clinical phenotype information in our study contains the following types of solid cancer: bladder cancer (n=61), prostate cancer (n=71), and lung cancer (n=163). Figure S1(see PDF) shows flowchart of creating analysis datasets for three cancer types. Each control group was created by randomly sampling of 100 samples from the group of non-disease samples (n=723) with clonal mosaicism, regardless of disease type, after adjusting for gender and age. For the lung cancer analysis dataset, 100 samples were randomly sampled from lung cancer patients. The maximum sample size of the analysis dataset was set to 200 because the calculation of similarity for all patient pairs requires massive analyses of 43,364 terms of all sub-GO, which is very computationally intensive. We annotate each sample with GO terms using biomaRt (version 3.13) [16] in the R environment from the position information, start and end in abnormal region. In total, case and control groups were combined to create each analysis datasets for the three cancer types in Sub-GOFA.

### Comparison with conventional statistical methods:

Two conventional statistical methods were used to compare the analysis performance of GO terms. They are (1) Fisher's exact test for gene symbols and (2) Fisher's exact test for GO terms. The FDR thresholds of each method, including Sub-GOFA, were set to the same value, and the detection performance was evaluated by comparing the detection ratio of cancer-associated genes while changing the FDR threshold values. The cancer-associated genes were extracted from DisGeNET [17], which is known as a platform containing the largest collection of genes and variants involved in human diseases.

## Results & Discussion:

**Figure 2:**
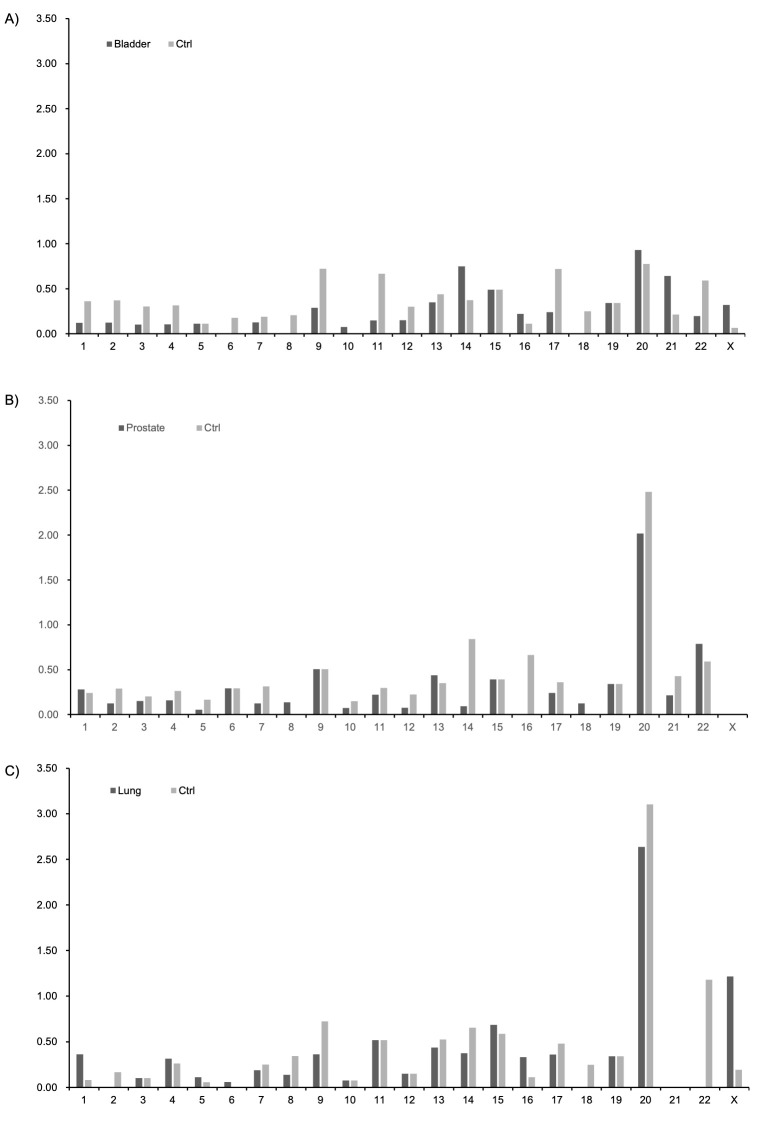
Comparison of case (cancer-patients) and ctrl (non-patients) for the frequency of clonal mosaicism at 1000 Mb in each chromosome.

Table S1 shows the number and proportion of males and females in the case and control groups, as well as the mean and standard deviation of age (supplementary material - see PDF).The analysis dataset does not show a significant difference in gender and age between the case and control groups under clonal mosaicism. In bladder cancer's case groups, 21.3% of the patients were males, 78.7% were females, and the age was 69.3 ± 6.71. Prostate cancer is a male cancer, and the age of the case group was 71.01 ± 6.72.In lung cancer's case groups, 52.1% were males, 47.8%were females, and the age was 67.25 ± 9.5.The control groups in the three cancer types of analysis dataset did not differ significantly from the case groups in terms of gender ratio and age. In addition, Figure 2 also shows a comparison of the frequency of clonal mosaicism events at 1000 Mb for each chromosome in the case and control groups in those age and gender-adjusted analysis dataset.

Sub-GOFA has superior performance in detecting cancer-associated genes compared to conventional statistical methods. We evaluated the performance of Sub-GOFA by comparing the detection ratio of lung cancer-associated genes obtained from DisGeNET with different FDR thresholds of Sub-GOFA and conventional statistical methods, Fisher's exact test for gene symbol and for GO, in the lung analysis dataset(Figure 1B).The ratio of lung cancer-associated genes detected by Sub-GOFA increased when strict FDR threshold values wereapplied, from 6.74% at FDR value of 0.5 to 12.9% at FDR value of 0.1. On the other hand, Fisher's exact test for GO showed a slight increase from 6.55% at an FDR value of 0.5 to 6.86% at FDR value of 0.1.Fisher's exact test for gene symbols was 2.75% at FDR value of 0.5, and all gene symbols were rejected at FDR value of 0.4 and later, resulting in "not applicable" (NA). Sub-GOFA is a genetic functional analysis that also considers low-frequency GO terms.Figure 1C shows a plot of the frequency and density of GO terms calculated by Sub-GOFA and Fisher's exact test for GO for lung cancer at FDR value of 0.3. The Sub-GOFA plot shows peaks between 0 and 20. On the other hand, the plot of Fisher's exact test for GO peaks between 20 and 40. Sub-GOFA handled more low-frequency GO terms and detected a higher ratio of lung cancer-associated genes compared to the conventional method.

**Figure 3:**
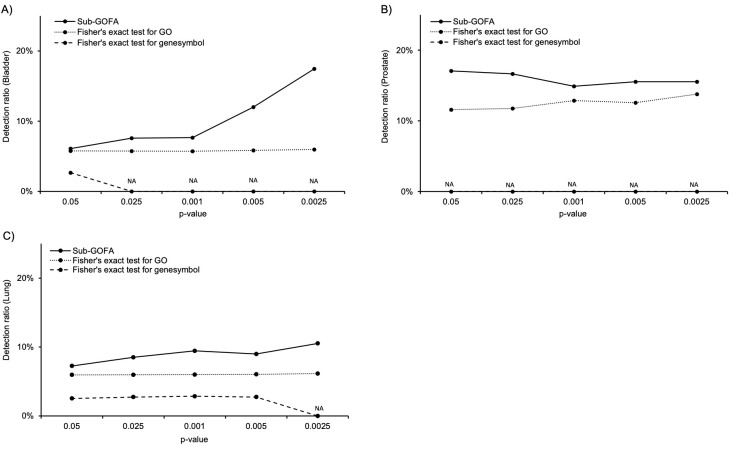
Comparison of detection ratio of cancer-associated genes among Sub-GOFA, Fisher's exact test for GO, and Fisher's exact test for gene symbols.

For all three types of cancers (bladder, prostate and lung cancer), Sub-GOFA showed higher detection performance of cancer-associated genes than the conventional methods even when p-value was set as the threshold. Since the FDR of Sub-GOFA did not show a significant difference for bladder cancer and prostate cancer, we validated the performance of Sub-GOFA and conventional statistical methods by comparing the detection ratio of cancer-associated genes at different p-value thresholds. For all three cancer types, Sub-GOFA showed the highest results compared to the conventional method at all setting threshold of p-value (Figure 3). Especially in bladder cancer and lung cancer, as the p-value was tightened, the detection ratio of cancer-associated genes increased. Sub-GOFA has the functionality to extract the genetic functional differences between lung cancer patients and non-patients under clonal mosaicism. Table 1(see PDF) shows the genetic functions with the 10 FDR values sorted in ascending order that obtained statistical differences between lung cancer patients and non-patients with the Sub-GOFA analysis. 'Protein localization to microtubule' (GO: 0035372, FDR = 0.0373) and ‘Negative regulation of histone methylation’ (GO: 0031061, FDR = 0.0398) were extracted from Sub-GOFA.GO:0035372contains MID1 (n=16, p-value=0.0209) genes, which have been reported as lung cancer-associated genes [18]. In a recent study, using blood-derived DNA methylation and gene expression profiles from a prospective lung cancer case-control study in women, 25 CpG lung cancer markers were identified prior to diagnosis [19]. Those existing research support the certainty of the genetic functional analysis by Sub-GOFA.

## Conclusion:

We describe a tool named Sub-GOFA for Sub-Gene Ontology function analysis in clonal mosaicism using semantic similarity. Sub-GOFA measures the semantic (logical) similarity among patients using the sub-GO network structures of various sizes segmented from the gene ontology (GO) for clustering analysis. The sub-GO's root-terms with significant differences are extracted as disease-associated genetic functions. Sub-GOFA selected a high ratio of cancer-associated genes under validation with acceptable threshold.

## References

[1] Schick UM (2013). PLoS ONE..

[2] Laurie CC (2012). Nature Genetics..

[3] Jacobs KB (2012). Nature Genetics..

[4] Machiela MJ (2015). American Journal of Human Genetics..

[5] MacHiela MJ (2016). Nature Communications..

[6] Schick UM (2013). PLoS ONE..

[7] Reina-Castillón J (2017). Blood Adv..

[8] Guo Y (2016). J Med Genet..

[9] Lareau CA (2019). Blood Adv..

[10] Zhao Y (2021). Nat Commun..

[11] Bonnefond A (2013). Nature Genetics..

[12] Harris MA (2004). Nucleic Acids Research..

[13] Mazandu GK (2017). Briefings in bioinformatics..

[14] https://dblp.org/.

[15] Greene D (2017). Bioinformatics..

[16] Durinck S (2009). Nature Protocols..

[17] Piñero J (2017). Nucleic Acids Research..

[18] Zhang L (2018). Journal of cancer research and clinical oncology..

[19] Sandanger TM (2018). Scientific Reports..

